# An unusual case of recurrent acute pancreatitis successfully treated via precut papillotomy (with video)

**DOI:** 10.1097/eus.0000000000000118

**Published:** 2025-07-01

**Authors:** Shi-Han Chen, Jia-Su Li, Wei Zhu, Yan Chen, Jie Chen

**Affiliations:** 1 School of Medicine, Jiangsu University, Zhenjiang, Jiangsu Province, China; 2 Department of Gastroenterology, Changhai Hospital, Naval Medical University, Shanghai, China.

Here we report a rare case of recurrent acute pancreatitis in a 34-year-old man caused by pancreatic stone impaction at the minor papilla rather than pancreas divisum (PD). Initial magnetic resonance cholangiopancreatography identified PD without pancreatic stones [Figure 1], but subsequent EUS revealed a protruding minor papilla and complete PD, with a 5 × 3-mm pancreatic stone impacted at the orifice of the dorsal pancreatic duct and diffuse ductal dilation [Figure 2]. Cannulation of minor papilla under endoscopic retrograde cholangiopancreatography failed, and the whitish stone was found embedded within the minor papilla [Figure 3]. A dual-knife papillotomy on minor papilla at the 12-o’clock position was used, exposing the impacted pancreatolith [Figure 4]. Precut papillotomy of minor papilla in the 4-o’clock direction was additionally performed, and the stone was successfully removed [Video 1]. The dorsal pancreatic duct was then cannulated easily, and a pancreatic stent was inserted into the duct for drainage. No postoperative complications occurred. Over 6 months of follow-up, the patient’s quality of life remained good.

**Figure 1:**
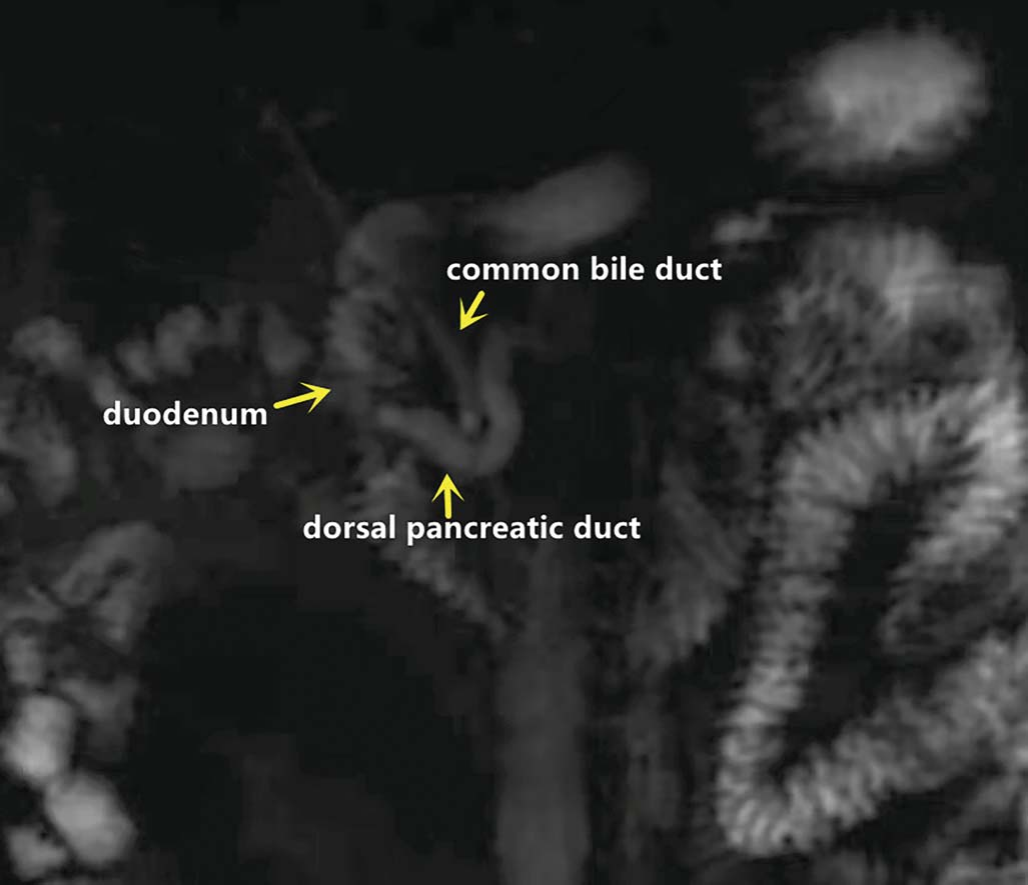
Magnetic resonance cholangiopancreatography showing pancreatic divisum and dorsal pancreatic duct dilatation but no evidence of pancreatic stone impaction at the orifice of dorsal pancreatic duct.

**Figure 2:**
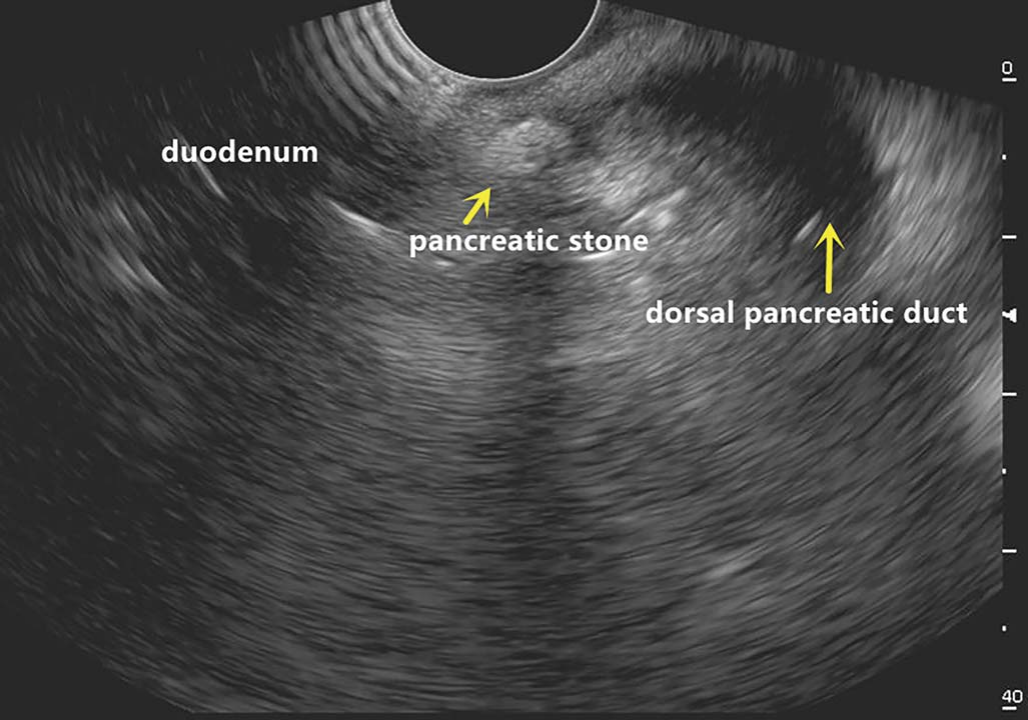
A pancreatic stone impacted at the orifice of the dorsal pancreatic duct with associated dilation of the whole duct.

**Figure 3:**
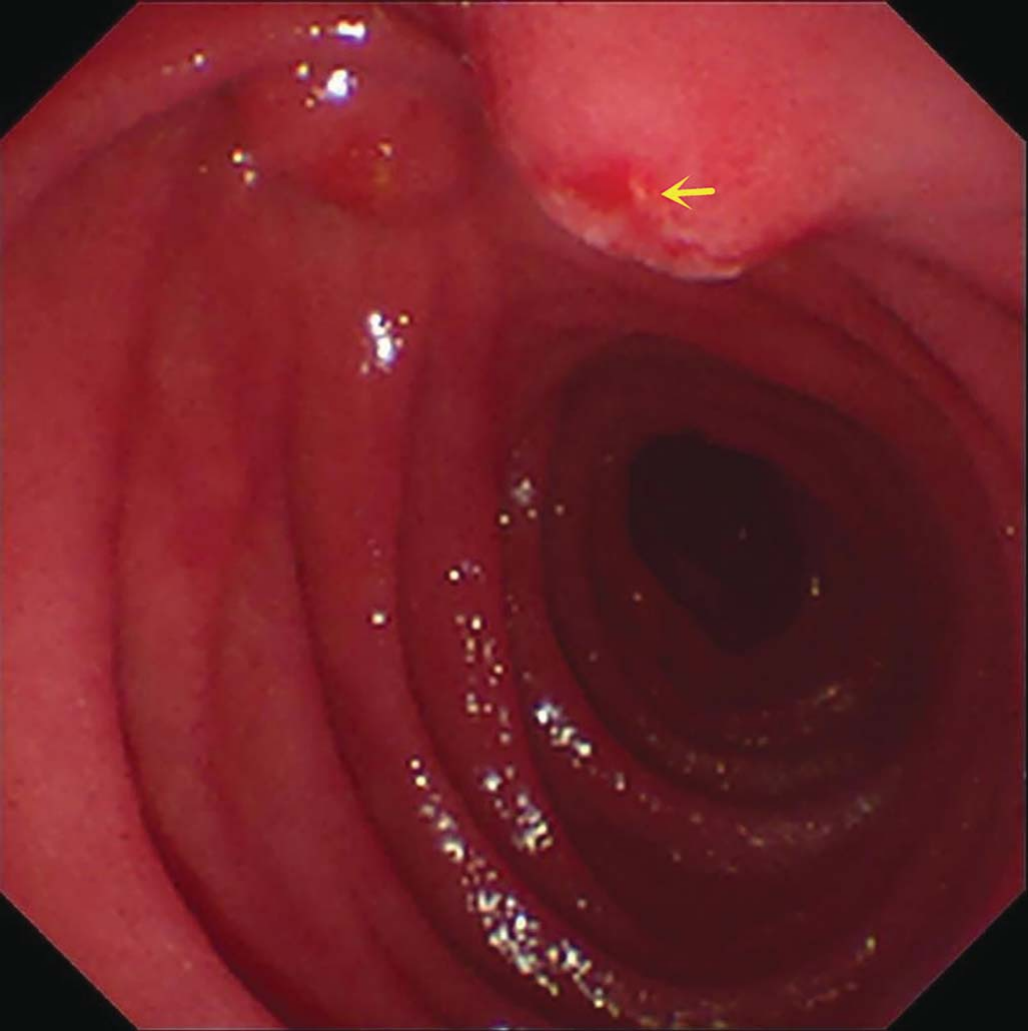
Endoscopic views of the stone extraction showing whitish pancreatolith (yellow arrows) trapped in the minor papilla.

**Figure 4:**
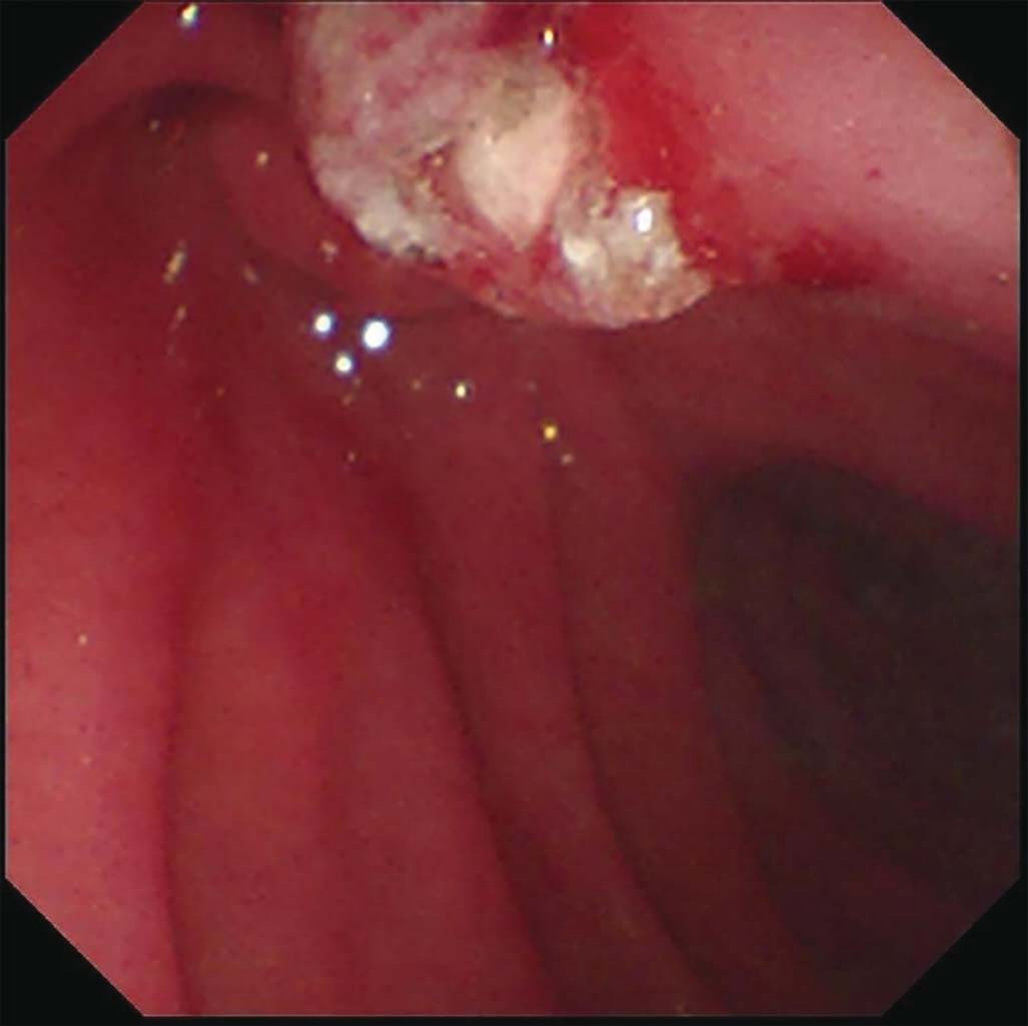
The stone exposed after dual-knife cutting in the 12-oʼclock direction.

This case also highlights that EUS has a superior diagnostic accuracy over magnetic resonance cholangiopancreatography for recurrent acute pancreatitis etiologies,^[1]^ and the dual-knife precut papillotomy technique is safe and effective in such clinical conditions.^[2]^

## Supplementary Videos

Video 1. Endoscopic extraction of a pancreatic stone encased in the minor papilla using dual-knife precut papillotomy technique allowing successful cannulation of the dorsal pancreatic duct. Videos are available only at the official website of the journal (www.eusjournal.com).

## Acknowledgments

None.

## Source of Funding

Dr Yan Chen is supported by the National Natural Science Foundation of China (No. 82200718).

## Ethical Approval

This case was conducted in accordance with the ethical standards described in the latest revision of the Declaration of Helsinki.

## Informed Consent

Informed consent for patient participation and publication was received from the patient.

## Conflicts of interest

The authors declare that they have no financial conflict of interest with regard to the content of this report.

## Author Contributions

Yan Chen and Jie Chen did the concept and design. Yan Chen did the acquisition of data. Shi-Han Chen and Jia-Su Li performed the analysis and interpretation of data. Shi-Han Chen and Jia-Su Li wrote and reviewed the manuscript. Wei Zhu, Yan Chen, and Jie Chen reviewed the manuscript and performed study supervision.

## Data Availability Statement

All data relevant to the case are included in the article.
